# Exploring proteomic immunoprofiles: common neurological and immunological pathways in multiple sclerosis and type 1 diabetes mellitus

**DOI:** 10.1186/s10020-025-01084-x

**Published:** 2025-02-03

**Authors:** Fátima Cano-Cano, Almudena Lara-Barea, Álvaro Javier Cruz-Gómez, Francisco Martín-Loro, Laura Gómez-Jaramillo, María Carmen González-Montelongo, María Mar Roca-Rodríguez, Lucía Beltrán-Camacho, Lucía Forero, Javier J. González-Rosa, Mª Carmen Durán-Ruiz, Ana I. Arroba, Manuel Aguilar-Diosdado

**Affiliations:** 1 Diabetes Mellitus Laboratory, Institute of Research and Biomedical Innovation of Cadiz (INiBICA), Cádiz, Spain; 2 Endocrinology and Metabolism Department, University Hospital Puerta del Mar, Cádiz, Spain; 3 Psychology Department, Institute of Research and Biomedical Innovation of Cadiz (INiBICA), University of Cadiz, Cádiz, Spain; 4 Biomedicine, Biotechnology and Public Health Department, Science Faculty, Biomedical Research and Innovation Institute of Cadiz (INIBICA), Cádiz University, Cádiz, Spain; 5 Neurology Department, Spain. Institute of Research and Biomedical Innovation of Cadiz (INiBICA), Puerta del Mar University Hospital, Cádiz, Spain

## Abstract

**Background:**

Interest in the study of type 1 diabetes mellitus (T1DM) and multiple sclerosis (MS) has increased because of their significant negative impact on the patient quality of life and the profound implications for the health care system. Although the clinical symptoms of T1DM differ from those of MS, such as pancreatic β-cell failure in T1DM and demyelination in the central nervous system (CNS) in MS, both pathologies are considered as autoimmune-related diseases with shared pathogenic pathways, which include autophagy, inflammation and degeneration, among others. Considering the challenges in obtaining pancreatic β-cells and CNS tissue from patients with T1DM and MS, respectively, it is fundamental to explore alternative methods for evaluating disease status. Proteomic analysis of peripheral blood mononuclear cells (PBMCs) is an ideal approach for identifying novel and potential biomarkers for both autoimmune diseases.

**Methods:**

We conducted a proteomic analysis of PBMCs from patients with T1DM and relapsing remitting Multiple Sclerosis (herein forth MS) patients (n = 9 per condition), using a label-free quantitative proteomics approach. The patients were diagnosed following the American Diabetes Association (ADA) criteria for T1DM and McDonald criteria for MS respectively, and were aged over 18 years and more than 2 years from the onset respectively.

**Results:**

A total of 2476 proteins were differentially expressed in PBMCs from patients with T1DM and MS patients compared with those form healthy controls (H). Predictive analysis highlighted 15 common proteins, up- or downregulated in PBMCs from patients with T1DM and MS patients *vs.* healthy controls, involved in the immune system activity (BTF3, TTR, CD59, CSTB), diseases of the neuronal system (TTR), signal transduction (STMN1, LAMTOR5), metabolism of nucleotides (RPS21), proteins (TTR, ENAM, CD59, RPS21, SRP9) and RNA (SRSF10, RPS21). In addition, this study revealed both shared and distinct molecular patterns between the two conditions.

**Conclusions:**

Compared with H, patients with T1DM and MS presented a specific expression pattern of common proteins has been identified. This pattern underscores the shared mechanisms involved in their immune responses and neurological complications, alongside dysregulation of the autophagy pathway. Notably, CSTB has emerged as a differential biomarker, distinguishing between these two autoimmune diseases.

**Supplementary Information:**

The online version contains supplementary material available at 10.1186/s10020-025-01084-x.

## Introduction

Type 1 diabetes mellitus (T1DM) and multiple sclerosis (MS) are severe autoimmune diseases with strong negative impacts on patients and profound implications for the health care system and society at large. Interest in the study of both diseases has increased, owing to the rapid increase in the prevalence of these pathologies over the past decades (Maahs et al. 2010; Walton et al. 2020; Gregory et al. 2022). Some studies have demonstrated that T1DM and MS share certain features and involve organ-specific mechanisms affecting various tissue targets (Pozzilli et al. 2022). In the case of T1DM, the immune system attacks pancreatic β-cells, leading to a failure in normal insulin production and eventually affecting glucose homeostasis (DiMeglio et al. 2018). Glucose regulation in these patients, which can be monitored by measuring HbA1c levels, is crucial in the progression of the inflammatory process and, consequently, in β-cell destruction (Bending et al. 2012) and the development of future peripheral complications, such as neurological complications or diabetic retinopathy among others (Melendez-Ramirez et al. 2010; Galiero et al. 2023; Perais et al. 2023). In MS, the autoimmune process induces a reactive response against antigenic elements of the central nervous system (CNS), leading to substantial disability in most patients (Cotsapas et al. 2018), which can be monitored by the Expanded Disability Status Scale (EDSS) and it is also related to the inflammatory process (Mungan et al. 2023). Previous data have provided evidence of the connection between demyelination, tissue injury and inflammation in all states of MS (Lassmann 2018).

The use of several omics technologies, such as high-throughput proteomics, constitutes an optimal approach to explore novel biomarkers, providing an enhanced understanding of disease mechanisms, insights into a etiology, and multifactorial pathophysiological processes (Zhi et al. 2011; Del Boccio et al. 2016). These advancements could contribute significantly to the development of therapeutic tools. Studies on the molecular basis of both autoimmune diseases, T1DM and MS, have demonstrated the involvement of various pathways, including autophagy, inflammation and degeneration, among others (Bending et al. 2012; Ruiz et al. 2019; Canet et al. 2022; Al-kuraishy et al. 2024). Evidence supports the roles of similar pathways and comparable responses that contribute to the pathogenic mechanisms of the diseases (Handel et al. 2009; Pozzilli et al. 2022).

Given the inherent difficulty in obtaining pancreatic β-cells from patients with T1DM and CNS tissue from patients with MS, there is a pressing need to develop noninvasive sampling techniques capable of accurately reflecting status. Because immune cells initiate the autoimmune and inflammatory processes against the corresponding target organs, the use of peripheral blood mononuclear cells (PBMCs) has emerged as ideal candidates for identifying new and potential biomarkers for both T1DM and MS. In addition, studying the proteomic signatures of both diseases could uncover underlying correlations, providing insight into their casualty.

In this study, a label-free quantitation (LFQ) proteomics approach was applied to identify common and differentially expressed proteins in PBMCs from patients with T1DM and MS. Additionally, we identified potential correlations and differences between these conditions. By leveraging this advanced analytical technique, we aimed to deepen our understanding of shared pathophysiological mechanisms and contribute to the discovery of new biomarkers.

## Methods

### Experimental design

**Fig. 1:**
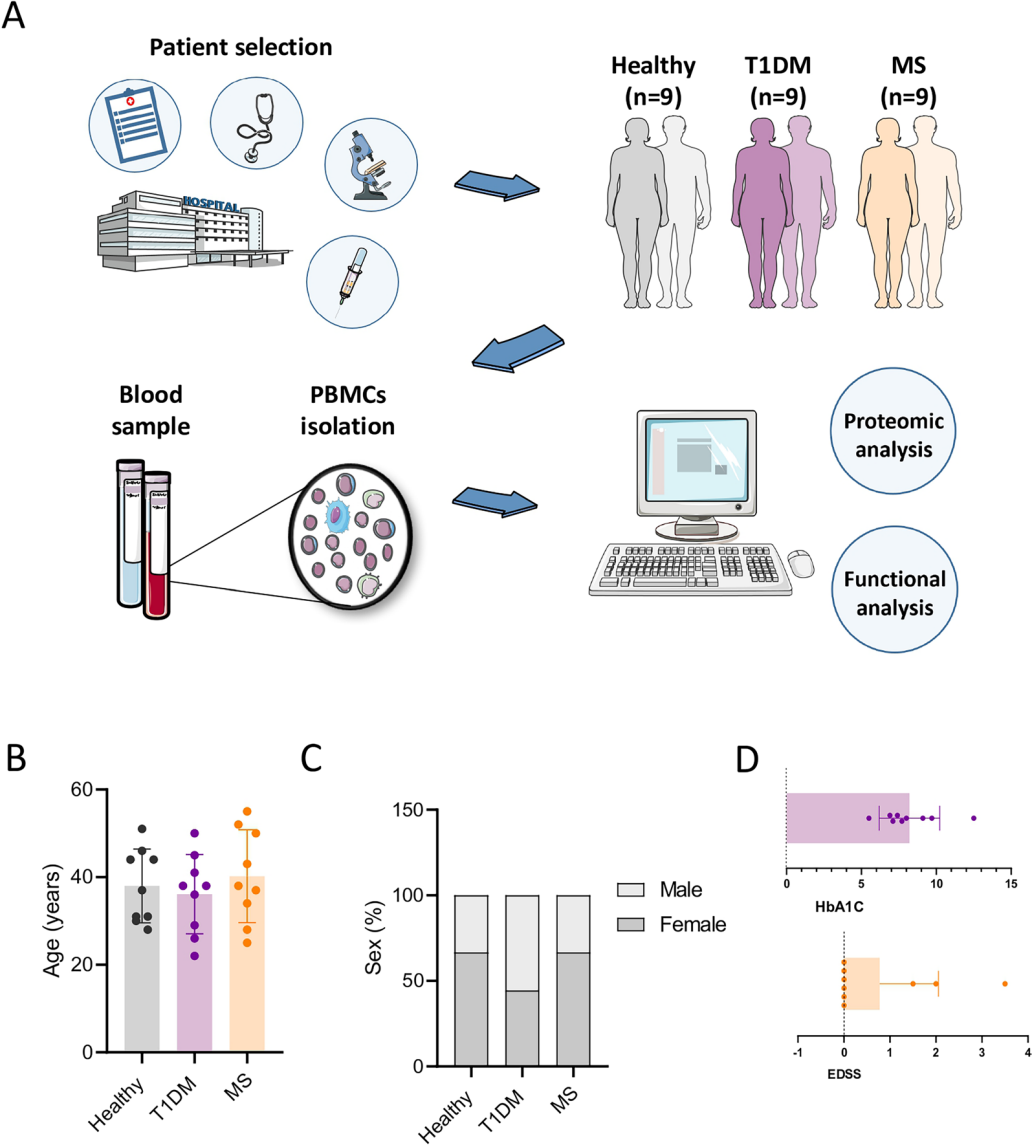
Experimental workflow and study population characteristics. **A** Schematic representation of the experimental assay. Healthy volunteers, patients with T1DM and MS were selected according to inclusion criteria. PBMCs were isolated from blood samples of each participant (n = 9 per condition). Finally, a LFQ proteomic approach was applied, together with bioinformatics analysis (statistic and functional classification) to identify differentially expressed proteins between groups. Graphical representation of donors (**B**) age, **C** gender and **D** HbA1c (up), EDSS (down) measures. HbA1c, glycosylated hemoglobin. EDSS, Expanded Disability Status Scale

An overview of the experimental workflow is shown in Fig. 1A.

### Study participants

In this study, 18 patients who were diagnosed with T1DM or relapsing–remitting Multiple Sclerosis (henceforth MS, n = 9 per condition) were recruited between 2020 and 2022 from Puerta del Mar University Hospital of Cadiz. In addition, another 9 volunteers from the same area, age and ethnicity as the patients, with no history of neurological, psychiatric or immunological diseases, were also recruited as a healthy group (H). Some of the characteristics of the study population (sex and age) are graphically represented in Figs. 1B, C.

The patient recruitment process was carried out by qualified neurologists and endocrinologists from the same hospital, following the most widely used diagnostic criteria at the time of diagnosis: the ADA criteria (Care and Suppl 2021) for T1DM and the McDonald criteria (Polman et al. 2011) for MS diagnosis. The following inclusion criteria were used for patients with T1DM: aged over 18 years; more than 2 years from disease onset; positive results for glutamic acid decarboxylase and tyrosine phosphatase auto-antibodies; and a continuous insulin treatment, with adequate glucose management, as reflected the HbA1c levels (Fig. 1D). The criteria for patients with MS were as follows: aged over 18 years; between 2 and 7 years from disease onset, experiencing mild physical disability (Fig. 1D); and having been free from relapses and steroid treatment for at least 2 months prior to the study, assuming the remission phase. In patients with MS, blood samples were routinely performed in the middle or at the end of their respective treatment cycles.

### PBMC isolation

Peripheral blood samples were collected in EDTA tubes, and PBMCs were isolated by a standard density gradient using Histopaquet 1077 (Sigma-Aldrich, Missouri, USA). Briefly, whole blood was extracted from EDTA tubes, diluted 1:1 in phosphate-buffered saline (PBS) and carefully layered over the same volume of Histopaquet. The tubes were centrifuged for 25 min at 1900 rpm with slow acceleration and no break to avoid disrupting the layers. After centrifugation, the interface layer was harvested and transferred to a new tube and the cells were washed twice in PBS (centrifugation; 10 min at 1500 rpm). The cells were counted using Trypan blue staining, ensuring a high viability rate (96–98%) at the time of freezing. In the last centrifugation, the supernatant was removed and the resulting cell pellet was snap-frozen and stored at -80°C until protein extraction.

### Proteomic analysis

For the proteomic analysis a volume of 250 μL of extraction buffer (7 M urea, 2 M thiourea, 0.4% CHAPS, 200 mM DTT) was added to each pellet for cell lysis and protein extraction. The samples were sonicated and centrifuged (13,000 rpm for 15 min), and the supernatants were transferred to another tube for further protein precipitation overnight with a 5X volume of acetone were at – 20 °C. Next, the samples were centrifuged (13,000 rpm for 15 min), the supernatants were discarded, and the pellets were resuspended in 100 μL of extraction buffer. The protein content was quantified following the Bradford method and 50 μg of protein from each sample were digested with trypsin (GOLD, Promega) following the FASP method with minor modifications (Wiśniewski et al. 2009).

The resulting peptides were speed vacuumed, resuspended in 0.1% trifluoroacetic and desalted and concentrated by using reverse-phase microcolumns (C18 OMIX, Agilent). Thus, 200 ng from each sample was loaded on an EVOSEP ONE (Evosep) coupled online to a PASEF powered Tims tof Pro (Bruker) tandem mass spectrometer. Data-dependent acquisition (DDA-PASEF) was applied with the 30 SPD method.

The raw mass spectra datasets were analysed using free MaxQuant (v1.6) software for protein identification and quantification (Cox and Mann 2008). To identify differential proteins between conditions, Perseus software (Tyanova et al. 2016) (https://www.maxquant.org/perseus/) was then employed to carry out LFQ analysis, considering proteins identified with at least one unique peptide at an FDR of 1% (PSM-level).

### Data processing and statistical rationale

Protein identification and quantification were conducted using PEAKS software (Bioinformatics Solutions Inc. in Waterloo, CA, USA). Searches were executed against a database that included canonical human UniProt/Swissprot entries, excluding isoforms. The precursor and fragment tolerances were set at 20 ppm and 0.05 Da, respectively. The PEAKS Q module within the PEAKS software was utilized for area-based label-free protein quantification.

The data were uploaded onto the Perseus platform (Tyanova et al. 2016) for further analysis. The data were log2 transformed, 70% valid values were filtered, and missing values were imputed from a normal distribution and categorically annotated to define conditions. We also used filter rows based on categorical columns to eliminate proteins identified only by site, reverse and potential contaminants. Two-sample student’s t-test for differential expression analysis were used with a p-value truncation (0.05 threshold p-value). The different protein expression levels were evaluated by comparing the healthy volunteer group against the T1DM and RRMS patient group, and comparing the patient groups against each other to determine the possible differences between the diseases. To discriminate between differentially expressed proteins (DEPs) between groups (patients with T1DM or RRMS and healthy controls), the p-value was set at < 0.05. Further proteins were considered as significantly up- or downregulated when − log 10 (p-value) > 1.3 and the log2 (fold change) rates) were > 1 for up-regulated or < − 1 for downregulated proteins, respectively. ROC curve analysis was used to evaluate the predictive value of selected proteins by using SPSS software, indicating sensitivity and specificity percentages, the AUC and 95% confidence intervals. A p-value < 0.05 was considered to indicate statistical significance.

Additional data processing and graphing were performed using Prism 8, R, Perseus, Circos and the image repository Smart Medical Art. The functional roles of proteins were analysed by using Ingenuity Pathway Analysis (IPA) (Qiagen) and Reactome (https://reactome.org/) software, String and functional protein association networks were constructed in STRING software (https://string-db.org/). The functional analysis was carried out by considering the classification made in IPA software of canonical pathways and disease and function classifications.

## Results

**Fig. 2:**
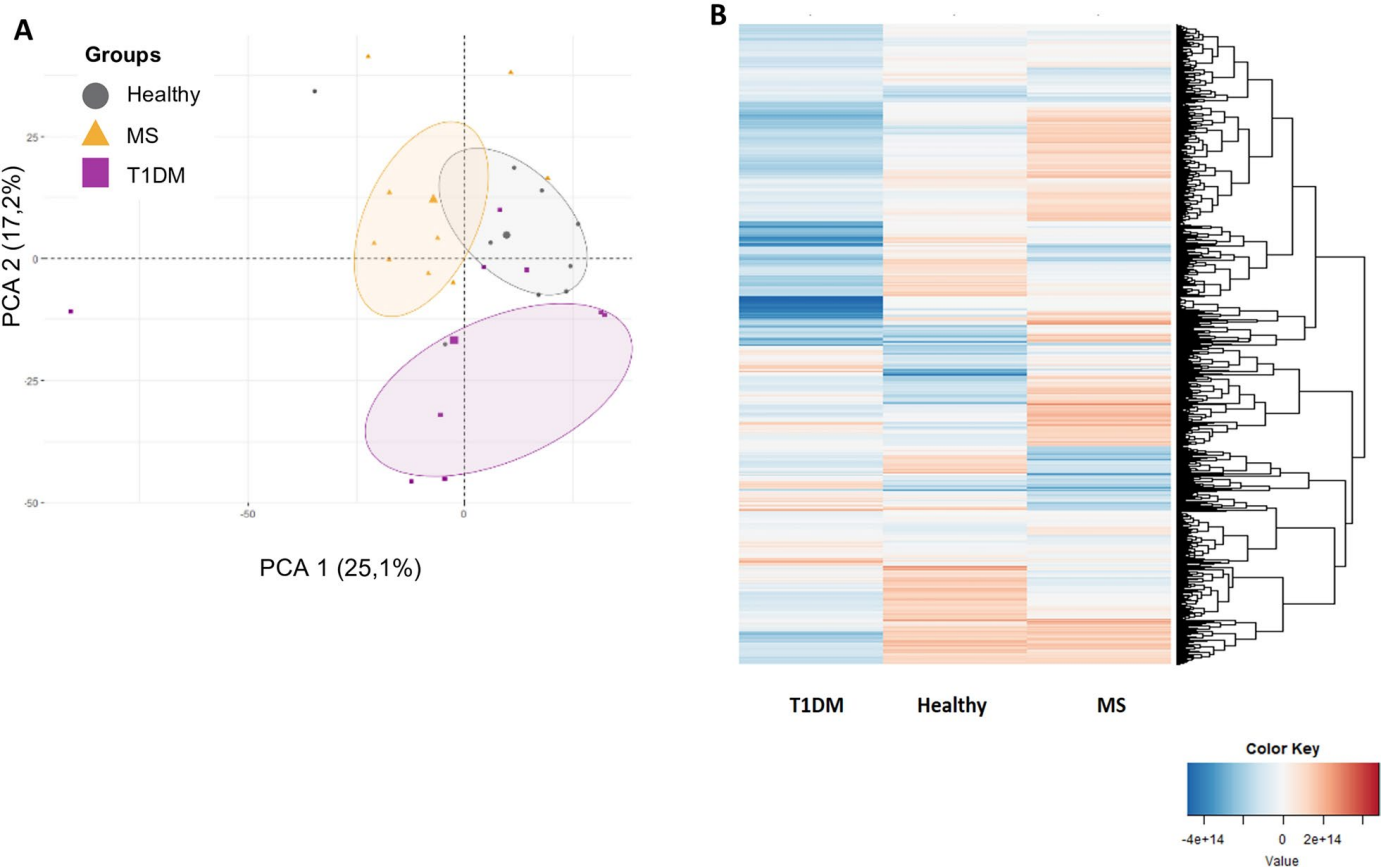
Proteomic study population clustering. (**A**) Principal component analysis and (**B**) Hierarchical cluster, representing the differential protein profiles

A LFQ proteomic analysis was carried out to investigate the differential proteomic expression of PBMCs from patients with T1DM and MS in the remission phase, and compared with healthy volunteers. On average, a total of 2476 proteins were identified, among the comparisons of patients with T1DM and MS *vs.* the healthy volunteers, resulting in an overall total of 674 proteins with significant differences (− log 10 p-value > 1.3) in all the comparisons. Notably, the proteome profiles of the groups of patients with T1DM and MS were different between from those of healthy controls, as indicated by the principal component analysis (Fig. 2A) and the hierarchical cluster (Fig. 2B) representations. Full information regarding identification and normalized protein intensities for T1DM and MS *vs.* healthy comparisons can be found in Supplementary Table S1.

### Differentially expressed proteins between T1DM and MS

**Fig. 3:**
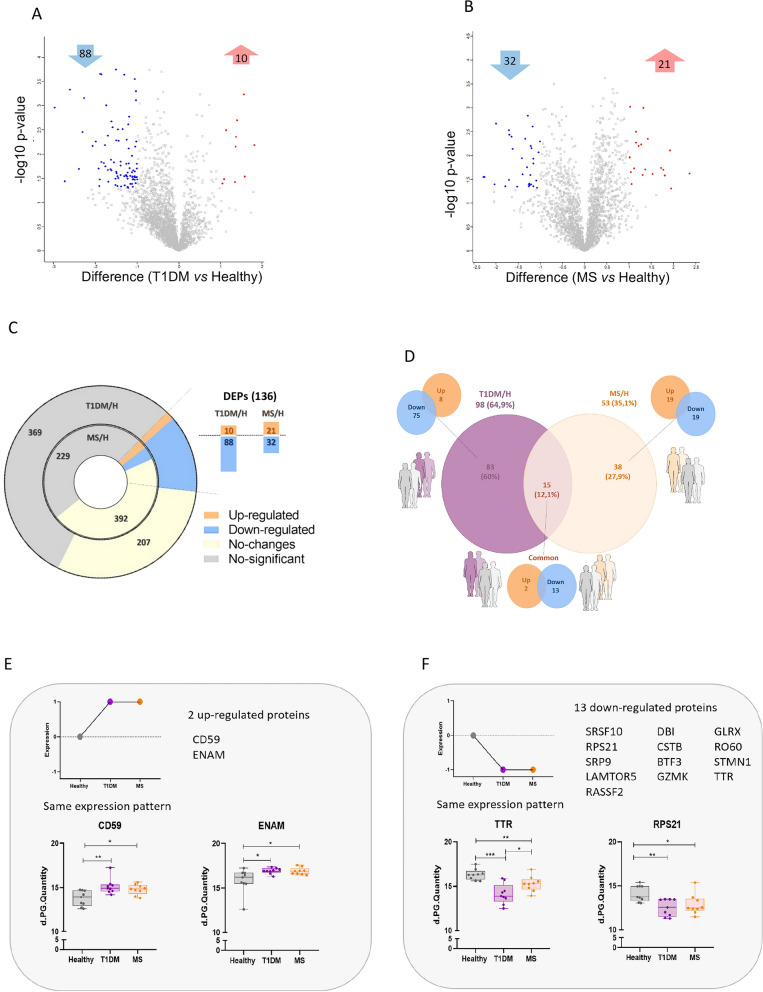
Protein changes in T1DM and MS. Volcano plots including the number of proteins up- (red) and down- (blue) regulated in (**A**) Patients with T1DM or (**B**) Patients with MS compared to Healthy controls. (**C**) Graphical representation of the number of differentially expressed proteins obtained from proteomic analysis in both diseases compared with healthy controls: Upregulated (orange) and downregulated (blue), DEPs without significant changes when compared with controls (yellow) and non-significant proteins (grey). (**D**) Venn’s diagram showing DEPs in T1DM (purple) and MS (light orange) and common DEPs in T1DM and MS *vs.* Healthy controls. A schematic representation of the changes observed for the common DEPs found (**E**) up- or (**F**) downregulated are shown, together with graphical representations of the Log2 (LFQ intensities) recorded for selected proteins identified through predictive analysis. *p-value < 0.05, p-value < 0.01. ***p-value < 0.001

Among the 674 DEPs, we focused on those with fold change rates ≥ 2, identifying in total 136 DEPs that were up- or downregulated between the patients with T1DM or MS and healthy controls group (H) (Fig. 3A–C). Notably, the number of DEPs found in patients with T1DM was greater (64.9%) than that found in patients with MS (35.1%), indicating that more proteins downregulated than upregulated in both cases. Additionally, the number of upregulated proteins in patients with MS *vs.* H was greater than that in patients with T1DM *vs.* H (21 *vs.* 10 proteins, respectively), whereas more downregulated proteins were found in patients with T1DM *vs.* H (88 downregulated) than in patients with MS *vs.* H (32 proteins) (Fig. 3C, D).

Furthermore, we found 15 common proteins (12.1%) with differential expression in both diseases compared with healthy controls, 2 of which were upregulated and 13 of which were downregulated; these proteins presented similar expression patterns in both cases (Fig. 3E, F). Additionally, the protein transthyretin (TTR) was downregulated in both the T1DM and MS *vs.* H groups, and it was also significantly downregulated in patients with T1DM compared with patients with MS (Fig. 3F).

Based on the information obtained from the Reactome Pathway Database (Supplementary Table 2), the 15 common DEPs identified in PBMCs from patients with T1DM and MS were involved in immune system activity (BTF3, TTR, CD59, CSTB), diseases of the neuronal system (TTR), signal transduction (STMN1, LAMTOR5), metabolism of nucleotides (RPS21), proteins (TTR, ENAM, CD59, RPS21, SRP9) and RNA (SRSF10, RPS21) (see Supplementary Table 2).

### Compared with healthy controls, differentially expressed proteins in patients with T1DM and MS are connected in several networks

**Fig. 4:**
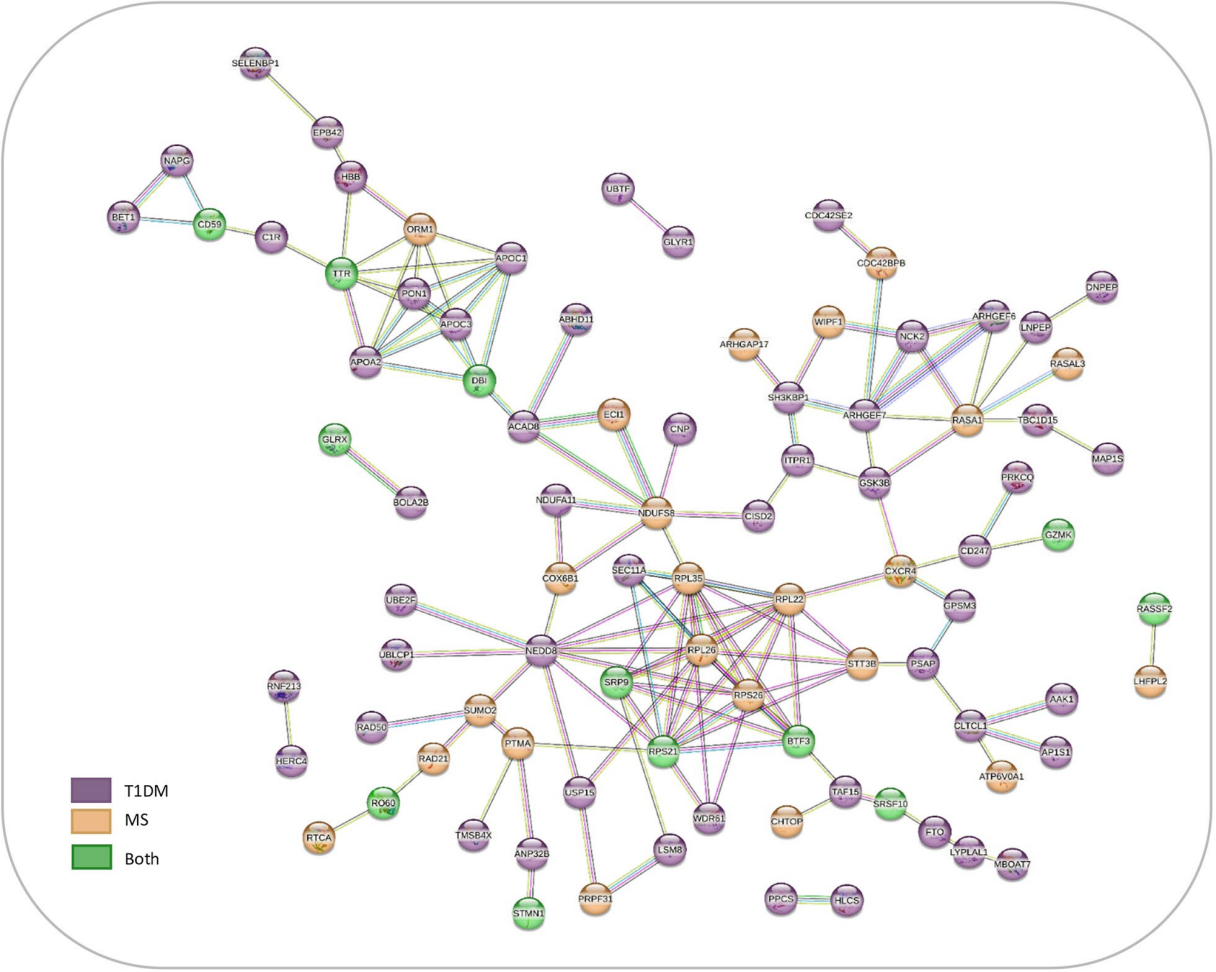
Relationship network analysis. The network represents the association between DEPs in patients with T1DM and MS *vs* healthy controls. Proteins are symbolized by network nodes, protein–protein connections are denoted by edges, and the thickness of the lines signifies the level of data support. The edges indicate both functional and physical protein associations. The minimum interaction score was set at 0.4. Disconnected nodes were not included, to provide a better view of the network. Nodes color represent, dysregulated proteins in T1DM (purple), MS (orange) and both diseases (green)

A relationship network analysis revealed strong connections between the 136 DEPs identified in both diseases, T1DM and MS, and compared to healthy controls (Fig. 4). Indeed, the analysis showed that nodes related to T1DM (purple) were closely connected with those related to MS (orange), instead of having two separate networks for each disease. Furthermore, some of the commonly altered proteins found in patients with T1DM and MS patients *vs.* H (green) were highly connected with dysregulated proteins in both comparisons (see Supplementary Table 3).

### T1DM and MS share several functional and disease-related pathways

**Fig. 5:**
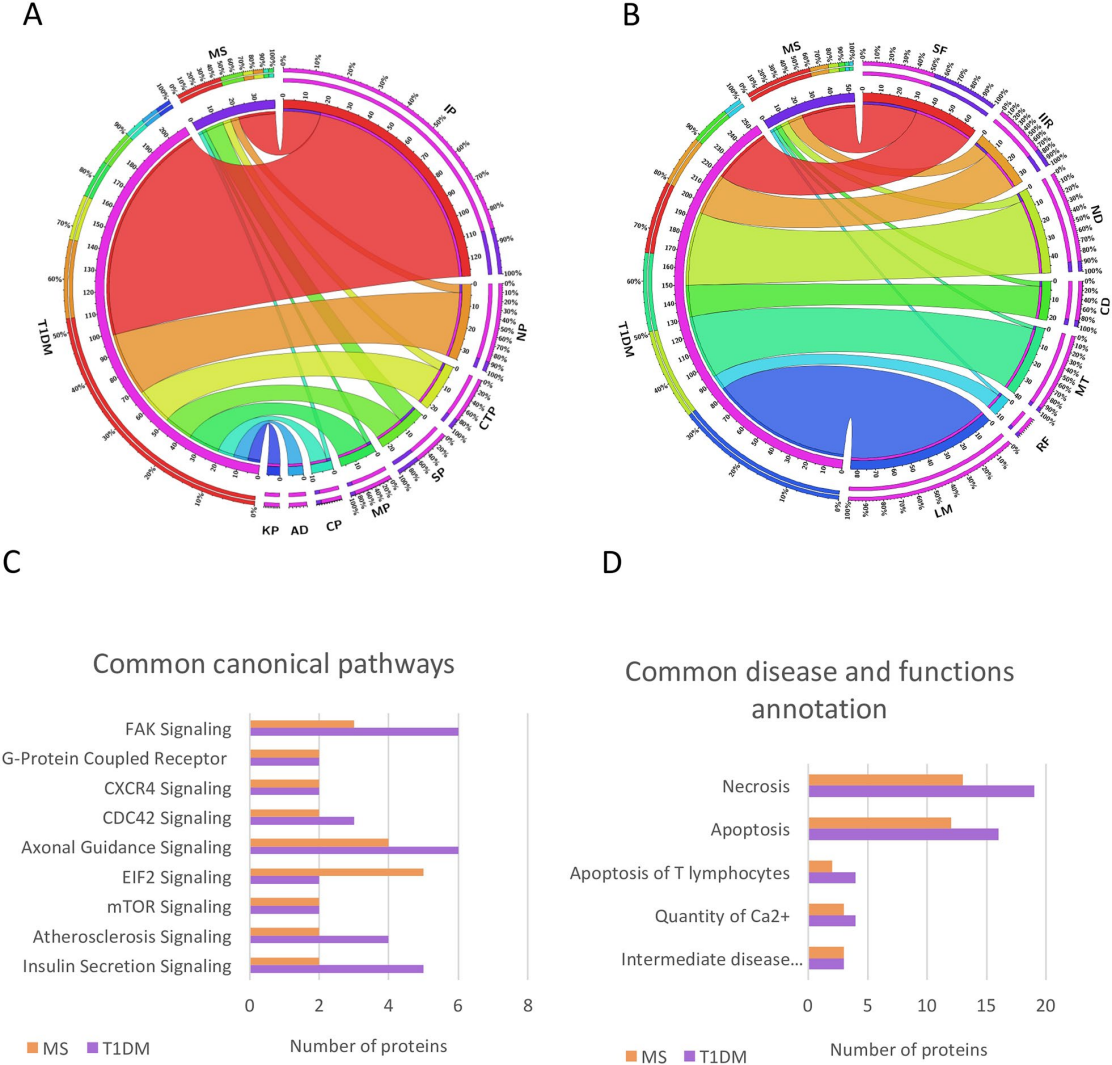
Functional analysis comparison between T1DM and MS.** A** The chart shows the correlations between the DEPs in patients with T1DM and MS (compared to Healthy controls) and different canonical pathways, according to Ingenuity functional assays: *IP* immunological pathways, *NP* neurological pathways, *CTP* cellular trafficking pathways, *SP* signaling pathways, *MP* metabolic pathways, *CP* cardiovascular pathways, *AD* Autoimmune diseases, *KP* kidney pathways. **B** The chart shows the correlations between the number of DEPs in T1DM and MS and the disease and functions found by ingenuity: *SF* signaling functions, *IIR* immune or inflammatory response, *ND* neurological disease, *CD* cardiovascular disease, *MT* membrane trafficking, *RF* renal function, *LM* lipid metabolism. **C** Common canonical pathways identified for T1DM and MS. Pathways with more than two significant proteins are represented. **D** Common disease and function annotation between patients with T1DM and MS. Pathways with more than two significant proteins are shown

According to the functional analysis performed with IPA, the protein changes detected in patients with T1DM or MS *vs.* healthy controls were related to several significant canonical pathways, such as immunological, neurological, and cellular trafficking or signalling pathways, among others (Fig. 5A).

Thus, many of the protein changes observed in both diseases were associated with the immune system (approximately 50% of them) including immunological or inflammatory functions, with a significant presence of proteins related to apoptosis or cell death of immune cells (mostly lymphocytes, ie. CD247, CD59, GSK3B, ITPR1, or PRKCQ) as shown in Table 1. In addition, other processes, such as membrane trafficking and metabolic or signalling pathways were also found to be commonly linked to the DEFs observed in patients with T1DM and MS compared with Healthy controls (Fig. 5A). Furthermore, in T1DM, many DEPs were related to lipid metabolism (33% T1DM, 0% MS), and in MS the majority of them were related to signalling functions (14% T1DM, 60% MS) (Fig. 5B). Notably, 10–15% of the altered proteins were also involved in neurological pathways in both diseases (Fig. 5A). Indeed, a greater number of proteins related to the neurological system were found in patients with T1DM than in patients with MS *vs.* H. In this sense, despite T1DM not being considered a neurological disorder, this disease has already been related to specific neurological complications and predisposes individuals to develop other neurological disorders (Chou et al. 2016; Ding et al. 2019; Jin et al. 2022). In T1DM, some of the terms annotated were related to myelin dysregulation (ARHGEF6, CNP, NADK2, PSAP, SLC25A12) and other neurological disorders, such as Alzheimer’s disease (i.e., POA2, APOC1, APOC3, CNP, GSK3B, PON1, PSAP, SELENBP1) or movement disorders (Table 2).

**Table 1:** Functional classification of protein changes due to immunological or inflammatory systems

Disease	Diseases or Functions Annotation	p-value	Molecules	Proteins
T1DM	Apoptosis of lymphocytes	3,96E-04	CD247↓, CD59↑, GSK3B↓, ITPR1↓, PRKCQ↓	5
	Apoptosis of leukocytes	5,62E-04	CD247↓, CD59↑, GSK3B↓, HBB↓, ITPR1↓, PRKCQ↓	6
	Complement activation	8,07E-04	C1R↓, CD59↑, RGCC↓	3
	Apoptosis of T lymphocytes	9,68E-04	CD247↓, CD59↑, GSK3B↓, PRKCQ↓	4
	Cell death of immune cells	1,16E-03	CD247↓, CD59↑, GSK3B↓, HBB↓, ITPR1↓, PRKCQ↓, SH3KBP1↓	7
MS	Apoptosis of leukemia cell lines	2,61E-03	CD59↑, CXCR4↑, RAD21↑, STMN1↓, VPS13A↑	5
	Cytotoxicity of lymphocytes	2,25E-02	CD59↑, WIPF1↓	2
	Apoptosis of T lymphocytes	2,39E-02	CD59↑, CXCR4↑	2

Protein classification was made with IPA software, showing the most probable functions of the proteins of interest, with at least two proteins related to the function. The table includes the p-values, molecule names and protein numbers included per disease or function annotation. Legend, ↑ up-regulated protein, ↓ down-regulated protein

**Table 2:** Functional classification of protein changes related with neurological system

Table Neurological functions				
Disease	Diseases or Functions Annotation	p-value	Molecules	Proteins
T1DM	Dysmyelination	3,90E-05	ARHGEF6↓, CNP↓, NADK2↓, PSAP↑, SLC25A12↓	5
	Leukodystrophy	2,28E-04	ARHGEF6↓, CNP↓, NADK2↓, PSAP↑	4
	Abnormal brain myelination	1,28E-03	CNP↓, PSAP↑, SLC25A12↓	3
	Hereditary myelin disorder	1,61E-03	ARHGEF6↓, NADK2↓, PSAP↑	3
	Hypomyelination of brain	6,41E-03	CNP↓, SLC25A12↓	2
	Alzheimer disease	6,85E-03	APOA2↓, APOC1↓, APOC3↓, C1R↓, CNP↓, GLRX↓, GSK3B↓, PON1↓, PSAP↑, SELENBP1↓	10
	Quantity of Ca2 +	2,16E-02	APOC3↓, CD59↑, F2RL3↑, ITPR1↓	4
	Outgrowth of neurites	3,60E-02	GSK3B↓, PSAP↑	2
	Movement Disorders	4,67E-02	AP1S1↓, ARHGEF7↓, CNP↓, CSTB↓, CYTH1↑, GSK3B↓, ITPR1↓, PON1↓, PSAP↑, SEC11A↓, STMN1↓	11
MS	Quantity of Ca2 +	2,01E-02	CD59↑, CXCR4↑, ORM1↑	3
	Formation of focal adhesions	2,25E-02	SLC9A1, STMN1↓	2

According to IPA database platform, some of the proteins changes were associated with several processes related to several neurological functions and/or related diseases, as shown. The table includes the p-values, molecule names and protein numbers included per disease or function annotation. Legend, ↑ up-regulated protein, ↓ down-regulated protein

The analysis also revealed common functional annotations between the two diseases, and there were several canonical pathways, such as those related to CXCR4, CDC42, FAK, axonal guidance or insulin secretion were shared among these pathologies (Fig. 5C). There were also some common annotations related to disease and function, most of which were related to cell death processes, such as necrosis or apoptosis, specifically the apoptosis of T cells (Fig. 5D).

### The predictive value of the TTR for the differential diagnosis of autoimmune diseases: T1DM and MS

To evaluate the diagnostic efficacies of specific proteins, receiver operating characteristic (ROC) curves were constructed utilizing the protein common or different protein sets (Table 3) previously identified in Fig. 3E–F. In the data obtained from the cohort, proteins such as TTR, SRSF10, RPS21, SRP9, DBI, BTF3, GZMK, RO60, STMN1, ENAM and CD59 had significant prognostic value in both the T1DM and MS groups. Furthermore, the proteins CSTB and RASSF2 presented a significant predictive value for differentiating between T1DM and MS (Table 3). These findings indicate that CSTB and RASSF2, as biomarkers, have a good diagnostic value for autoimmune disease and distinguishing between T1DM and MS, with a similar autoimmune profiles.

**Table 3:** ROC curves

Proteins	Healthy versus T1DM				Healthy versus MS				T1DM versus MS			
	AUC (95% CI)	Sensitivity (%)	Specificity (%)	p-value	AUC (95% CI)	Sensitivity (%)	Specificity (%)	p-value	AUC (95% CI)	Sensitivity (%)	Specificity (%)	p-value
TTR	0,95 (0,8573–1)	100	77,78	0,0013	0,8765 (0,6974–1)	100	77,78	0,0071	0,1975 (0–0,4228)	77,78	22,22	0,03051021
SRSF10	0,8641 (0,6819–1)	100	55,56	0,0092	0,8518 (0,6616–1)	100	55,56	0,0118	0,4074 (0,1317–0,6830)	55,56	33,33	0,50,780,065
RSP21	0,9012 (0,7535–1)	100	77,78	0,0041	0,8271 (0,6187–1)	100	77,78	0,0193	0,4567 (0,1714–0,7420)	77,78	44,44	0,75,727,775
SRP9	0,8518 (0,6758–1)	88,89	55,56	0,0118	0,9259 (0,8046–1)	77,78	77,78	0,0023	0,4938 (0,2005–0,7870)	77,78	55,56	0,96,478,398
DBI	1 (1–1)	100	100	0,0003	0,8271 (0,6237–1)	100	100	0,0193	0,2962 (0,0474–0,5451)	44,44	55,56	0,14,512,036
BTF3	0,9012 (0,7571–1)	88,89	77,78	0,0041	0,765 (0,5304–1)	88,89	77,78	0,0576	0,4691 (0,1899–0,7483)	55,56	44,44	0,8,252,829
GZMK	0,7654 (0,5417–0,9891)	77,78	66,67	0,0576	0,8395 (0,65,038–1)	77,78	66,67	0,0152	0,4444 (0,1590–0,7298)	44,44	44,44	0,69,110,222
RO60	0,9135 (0,7799–1)	100,00	77,78	0,0031	0,8518 (0,6409–1)	100	77,78	0,0118	0,6049 (0,3290–0,8808)	66,67	66,67	0,45,291,248
STMN1	0,8395 (0,6295–1)	88,89	88,89	0,0152	0,8888 (0,7392–1)	88,89	88,89	0,0054	0,679 (0,4055–0,9524)	77,78	77,78	0,20,041,107
CSTB	0,0493 (0–0,1426)	22,22	11,11	0,0013	0,2592 (0,0271–0,4913)	11,11	33,33	0,0851	0,7777 (0,5499–1)	77,78	77,78	0,04694473
LAMTOR5	0,1851 (0–0,4219)	22,22	11,11	0,0243	0,4197 (0,1211–0,71,834)	77,78	11,11	0,5660	0,6049 (0,3328–0,8770)	66,67	55,56	0,45,291,248
RASSF2	0,037 (0–0,1200)	11,11	33,33	0,0009	0,2839 (0,0268–0,5410)	11,11	33,33	0,1223	0,7654 (0,5417–0,9891)	66,67	77,78	0,05763072
GLRX	0,2098 (0–0,4344)	22,22	55,56	0,0380	0,1111 (0–0,2708)	22,22	55,56	0,0054	0,4197 (0,1428–0,6966)	44,44	55,56	0,56,599,215
ENAM	0,8395 (0,6370–1)	77,78	88,89	0,0152	0,8024 (0,5914–1)	77,78	88,89	0,0305	0,4444 (0,1608–0,7280)	44,44	55,56	0,69,110,222
CD59	0,8765 (0,7158–1)	88,89	66,67	0,0071	0,8271 (0,6370–1)	88,89	66,67	0,0193	0,395 (0,1268–0,6632)	44,44	44,44	0,45,291,248

Receiver operating characteristic (ROC) analysis of proteins in T1DM and MS versus Healthy individuals and T1DM versus MS comparisons of common dysregulated proteins between groups. AUC; Area Under the curve ROC and 95% confidence intervals

## Discussion

In this study, we have described the involvement of the immune system through common signalling pathways in both T1DM and MS, despite their differing pathophysiologies, providing a new perspective on the molecular basis underlying these diseases. The connections observed between the proteins involved and the mechanisms activated in both immunological and neurological contexts allow for the establishment of differential and/or similar profiles in the diagnosis and progression of T1DM and MS. In this study, we employed an LFQ proteomics approach to identify DEPs in PBMCs from individuals diagnosed with T1DM or MS compared with those from healthy controls. Furthermore, we have discerned the potential correlations and differences existing between these two conditions. Both autoimmune diseases presented a high number of common proteins involved in the development of T1DM and MS; however, our results have identified two specific proteins with significant profile changes compared with those in the healthy population, and with a potential diagnostic value for differentiating both pathologies: CSTB and RASSF2.

The co-occurrence of different autoimmune diseases has been a matter of interest in several studies as a way of understanding the autoimmune process (Cojocaru et al. 2010; Fidalgo et al. 2023). In the case of T1DM, there is a threefold greater risk of developing MS as a comorbidity than in the general population (Bechtold et al. 2014). This increased risk could be connected with the common relationship of both diseases with T-cell mediated autoimmunity. T-cell responses appear to be less organ-specific than might be anticipated from the two different conditions, as cross-reactivity between their tissues has been demonstrated. Indeed, T-cells from patients with T1DM present reactivity against pancreatic islet and CNS antigens, and this phenomenon takes place similarly in patients with MS (Winer et al. 2001; Banwell et al. 2008). This fact, combined with previous evidence (Marrosu et al. 2002; Zoledziewska et al. 2009; Pozzilli et al. 2022), clearly indicates the plausible correlation between the two diseases and how alterations in the immune system significantly impact them. The data analysed in this work reflect an established connection between the immune system modulation and neurological involvement. Furthermore, comprehending these correlations might contribute to explaining causality for both T1DM and MS. In this autoimmune and inflammatory context, it seems reasonable to study PBMCs, as precursors of immune cells, to unravel the underlying mechanisms involved.

In agreement with previous studies showing the existence of a differential transcriptional expression profile in PBMCs between healthy controls and those with T1DM and MS when analysed separately (Brynedal et al. 2010; Safari-Alighiarloo et al. 2017), our investigation revealed a discernible differential proteomic profile not only between healthy controls and individuals afflicted with either T1DM or MS but also between the two patient groups. Indeed, the results reflect a specific protein expression pattern for each pathological group, despite their similar autoimmune origin. These findings could contribute to the identification of potential protein biomarkers (CSTB and RASSF2) that are either shared between T1DM and MS or specific to each disease. Furthermore, patients with T1DM presented a greater number of DEPs than patients with MS did, with the former presenting nearly twice as many DEPs as the latter (98–53 proteins, respectively). This phenomenon may be linked to the disease, as evidenced by the observed discrepancy in the number of dysregulated genes within PBMCs during relapse or remission periods in MS in previous studies (Brynedal et al. 2010). These fluctuation periods have also been described in T1DM as part of the immunomodulatory process that occurs during β-cell destruction, resulting in a distinct signature throughout T1DM progression. Moreover, some investigations have suggested that this process contributes to the existence of a continuous relapsing remitting profile of β-cell mass and variations in the destructive autoreactive response (von Herrath et al. 2007; Van Belle et al. 2011; van Megen et al. 2017). The oscillations in the immunological and inflammatory processes during the course of each disease, could influence the abundance of DEPs present in PBMCs at each time point of these pathologies.

The analysis of common dysregulated proteins between both pathologies, revealed the relationships of those proteins with the immune system activity, diseases of the neuronal system, signal transduction, metabolism of nucleotides, proteins and RNA processing. With respect to immune system activity, we found a connection with two different pathways, interactions with the butyrophilin (BTN) family and neutrophil degranulation (Supplementary Table S2). The BTN family has been linked to both stimulatory and inhibitory effects on cells within the immune system, especially T lymphocytes (Malinowska et al. 2017). More specifically, BTF3 down-regulation (Fig. 3F) has been connected with the inhibition of transcription and protein synthesis in apoptotic K562 cells and is involved in the regulation of apoptosis in animal models (Jamil et al. 2015), suggesting that BFT3 downregulation could compromise cell viability in specific target organs to both T1DM and MS. The recruitment and activity of different immune cells during T1DM and MS contribute to disease development. Furthermore, the role of neutrophils has been proposed to be crucial in the onset and progression of both diseases, because of their capacity for degranulation and heightened production of reactive oxygen species (ROS) in target tissues (Huang et al. 2016; De Bondt et al. 2020). ROS production is related to the death of pancreatic β-cells in T1DM (Obeagu and Obeagu 2023), and is also being associated with demyelination and damage to astrocytes and axons in MS (Larochelle et al. 2011). There is substantial evidence of oxidative and nitrosative stress in patients with MS, as demonstrated by elevated serum levels of ascorbic acid, nitrites, and malondialdehyde compared with those in the healthy population. These findings suggest that increased lipid peroxidation is a consequence of exacerbated ROS production (Rispoli et al. 2021). Lipid peroxidation exerts its pathological effects by modifying specific proteins in patients with MS, which leads to the generation of autoantibodies against these lipid peroxidation-modified proteins (Gonzalo et al. 2012). Furthermore, oxidative and nitrosative stress have been associated with increased disability in patients with MS (Kallaur et al. 2017). In contrast, our results indicate the downregulation of RPS21, which may possibly indicate an activation of autophagy processes (Al-kuraishy et al. 2024), potentially linked to the remitting phase of MS.

Notably, we detected lower levels of TTR protein in PBMCs from patients with both diseases than in those from healthy controls. TTR plays a role in various neuronal processes, including the transport of retinol and thyroid hormones (Ueda 2022), which could be affected in both diseases (Lehmensiek et al. 2007; Forga et al. 2016). Furthermore, TTR has been shown to play roles in oligodendrocyte development and the process of myelination, by producing hypermyelination in TTR-null mice (Alshehri et al. 2020). Microstructural abnormalities in the white matter of the brain have been found in patients with T1DM, suggesting the presence of injury in myelinated fibres or axonal degeneration (Toprak et al. 2016; Muthulingam et al. 2022). TTR is involved in oligodendrocyte development and myelination processes (Alshehri et al. 2020), where its absence or low levels are associated with enhanced and faster remyelination. This implies that TTR might act as a modulator that, when absent, allows for improved remyelination (Pagnin et al. 2022), during the remission phase of patients with MS.

Notably, TTR levels were significantly lower in patients with T1DM than in patients with MS, underscoring the potential importance of this protein in the nervous system. This reduction in TTR could be associated with the prevalence of developing diabetic retinopathy (DR), and it has been proposed as a potential marker for the diagnosis and treatment of DR (Sun et al. 2022), justifying its crucial role in the development of neurological alterations.

Additionally, we observed alterations in the ERBB4 signalling pathway in both diseases (Supplementary Table 2). There is some evidence of reduced ERBB4 expression in the immune cells of patients with MS, suggesting that this protein is involved in the proliferation of oligodendrocyte progenitor cells, the differentiation of oligodendrocytes and remyelination (Tynyakov-Samra et al. 2011). However, evidence regarding the involvement of this pathway in typical neurological alterations in T1DM is currently limited. Furthermore, it is noteworthy that our analysis of pathways implicated in each disease, with respect to neurological functions, revealed several pathways linked with myelin dysregulation in T1DM (Table 2). These include dysmyelination, abnormal brain myelination or hypomyelination of the brain in T1DM, as well as axonal guidance in both patients with T1DM and MS (Fig. 5C). Although dysregulations in axonal guidance and myelin metabolism have been extensively studied in MS (Berg et al. 2017; Lemus et al. 2018), evidence in T1DM is limited. Therefore, further research is warranted to elucidate potential myelin-related dysregulations in T1DM.

Among the proteins associated with these pathways, ribosomal protein S21 (RPS21) protein, which is related to dysregulation of the translation process, was downregulated (Wang et al. 2020; Pöll et al. 2023). Notably, this protein is important not only because of its dysregulation in both diseases, but also because it presented the greatest number of connections (11 connections) in the network analysis. Moreover, the presence of numerous connections between proteins highlights their possible involvement in several pathways essential for normal cellular function. Specifically, RPS21 alteration could be related to alterations in several pathways, such as ER stress, and consequently, it may play a role in the regulation of autophagic processes in both diseases. The expression of this protein has not been studied before in either of the two pathologies, underscoring the importance of investigating its role in elucidating its implications in both.

Additionally, we analysed the altered pathways by incorporating all the dysregulated proteins in each disease separately. With respect to functional pathways, our results revealed that a high proportion of proteins were associated with lipid metabolism in T1DM, as shown in Fig. 5 (33% in T1DM, 0% in MS), whereas signalling functions predominated in MS (14% in T1DM, 60% in MS). Lipid metabolism is related to a switch in metabolic signatures in T-cells and macrophages (Catarino et al. 2020; Villoria-González et al. 2023) during differentiation and activation (Endo et al. 2022). Similarly, these processes may be implicated in both diseases, as has been previously studied in MS (Pompura et al. 2022). However, further evidence is needed to understand its role during the relapse and remission periods in MS and the subjacent mechanisms involved in T1DM. Notably, the primary signalling functions analysed were involved in survival and apoptotic processes. These results emphasize the critical role of controlling cell viability during the immune response in both chronic autoimmune diseases.

In addition, we observed alterations in several pathways, including mTORC1-mediated signalling, the cellular response to starvation and the GCN2 response to amino acid deficiency, all of which have been linked to autophagy activation (Hamasaki et al. 2005; Singh and Cuervo 2011; Masson 2019). Autophagy plays an important role in cell survival under certain conditions, such as inflammation, neurodegeneration and starvation (Chatterjee et al. 2019). Suggestions have been made that prolonged and excessive exposure to glucose and fatty acids might block the natural adaptive mechanisms, such as autophagy, within β-cells to protect themselves from the toxicity and stress associated with the T1DM environment (Marasco and Linnemann 2018). In the context of MS, neuronal loss may be linked to the normal function of neuronal autophagy, as well as other surrounding cells, such as microglia and oligodendrocytes. Both cell types are involved in myelin debris clearance and impaired remyelination, respectively, potentially contributing to neuronal death (Misrielal et al. 2020). In addition to autophagy dysregulation in target tissues, dysregulation of autophagy has also been identified in the PBMCs of both diseases (Canet et al. 2022; Al-kuraishy et al. 2024). This promotes autophagy activation in PBMCs, protecting them from an inflammatory environment (Chatterjee et al. 2019) and assisting in their survival through protein turnover associated with cell death (Botbol et al. 2016). The survival of these immune cells may trigger a sustained immune response. Moreover, we identified several dysregulated pathways associated with various stages of protein translation, potentially linked to the presence of endoplasmic reticulum (ER) stress in PBMCs, which plays a crucial role in autophagy activation(Deegan et al. 2013), such as EIF2 signalling. EIF2 has been associated with ER stress, as it prevents ribosome assembly, leading to the global downregulation of protein translation (B’chir et al. 2013). This finding supports the down-regulation of the RPS21 protein in our patients with T1DM and MS, potentially indicating a decrease in protein translation due to autophagy activation in PBMCs.

Moreover, our analysis revealed numerous dysregulated pathways associated with immune cell apoptosis and cell death, which are linked mainly to lymphocyte apoptosis. As mentioned previously, the activation of autophagy mitigates cellular stress, thereby preventing apoptosis and promoting cell survival. There is evidence of apoptosis in PBMCs in both patients with T1DM (Hu et al. 2020) and patients with MS (Mandel et al. 2012), supporting that increased tolerance to the apoptosis of autoimmune cells underlies the pathogenesis of these diseases.

## Study limitations

Some limitations should be considered. First, the influence of disease-modifying therapies (DMTs) in patients with MS represents a potential confounding factor. Different DMTs have varying mechanisms of action—ranging from immunomodulation to selective immune cell depletion—and may influence proteomic profiles (Oreja-Guevara et al. 2024). To mitigate long-lasting effects on the immune system, blood samples from MS patients were always obtained mid-to-end of of the corresponding treatment cycles, when a gradual reconstruction or complete recovery of the immune system is assumed. And we included patients with T1DM, who were not receiving immunomodulatory or immunosuppressive treatments, as a comparator group. This allowed us to identify proteins and pathways that remain consistently dysregulated under both conditions, potentially reflecting robust autoimmune-related mechanisms rather than treatment-induced effects. Second, our study is limited by a small sample size (n = 9 per group), which, although sufficient to reveal significant proteomic differences, may reduce the generalizability of our findings. Larger cohorts will be necessary to validate the identified biomarkers and ensure their reproducibility across diverse patient populations and clinical settings. Third, although bioinformatics tools such as Reactome and IPA enabled us to integrate and interpret the high volume of proteomic data, these analyses primarily highlight associations between proteins and specific pathways. Such tools indicate pathway involvement but do not provide information regarding pathway activation or suppression. Future experimental studies, including functional assays, are needed to delineate the orientation and impact of these pathways in autoimmune conditions such as MS and T1DM. Notwithstanding these drawbacks, our study may represent an important step toward for comprehending both common and disease-specific mechanisms in T1DM and MS. This study highlights key proteins and pathways that may serve as potential biomarkers of immune dysregulation and neuroinflammatory processes, laying the groundwork for future studies aimed at confirming and expanding upon these findings.

## Conclusions

During T1DM and MS, similar immune and neurological processes occur with distinct pathological implications and differential protein expression. The identification of specific expression patterns for common proteins in both autoimmune diseases suggest that the underlying mechanisms involved in the immune response are linked to the development of various neurological complications, accompanied by autophagy pathway dysregulation. Thus, our results revealed that two of the common proteins for T1DM and MS, CSTB and RASFF2, are potential biomarkers for differentiating between these autoimmune diseases.

## Supplementary Information

Supplementary material 1.

## Data Availability

Proteomic data that support the findings of this study have been deposited in the ProteomeXchange Consortium via PRIDE partner repository and in supplementary information files.

## Abbreviations

BTN: Butyrophilin
CNS: Central nervous system
CSTB: Cystatin B
DEPs: Differentially expressed proteins
EDSS: Expanded Disability Status Scale
ER: Endoplasmic reticulum
H: Healthy group
HbA1c: Glycosylated hemoglobin
LFQ: Label-free quantitation
MS: Multiple sclerosis
PBMCs: Peripheral blood mononuclear cells
PBS: Phosphate-buffered saline
RASSF2: Ras association domain family member 2
ROS: Reactive oxygen species
RPS21: Ribosomal protein S21
RRMS: Relapsing–remitting multiple sclerosis
T1DM: Type 1 diabetes mellitus
TTR: Transthyretin

## References

[CR1] Al-kuraishy HM, Jabir MS, Al-Gareeb AI, Saad HM, Batiha GES, Klionsky DJ. The beneficial role of autophagy in multiple sclerosis: yes or no? Autophagy. 2024;20:259–74. 10.1080/15548627.2023.2259281. 37712858 10.1080/15548627.2023.2259281 PMC10813579

[CR2] Alshehri B, Pagnin M, Lee JY, Petratos S, Richardson SJ. The role of transthyretin in oligodendrocyte development. Sci Rep. 2020;10:4189. 10.1038/s41598-020-60699-8. 32144308 10.1038/s41598-020-60699-8 PMC7060235

[CR3] B’chir W, Maurin A-C, Carraro V, Averous J, Jousse C, Muranishi Y, Parry L, Stepien G, Fafournoux P, Bruhat A. The eIF2α/ATF4 pathway is essential for stress-induced autophagy gene expression. Nucleic Acids Res. 2013;41:7683–99. 10.1093/nar/gkt563. 23804767 10.1093/nar/gkt563 PMC3763548

[CR4] Banwell B, Bar-Or A, Cheung R, Kennedy J, Krupp LB, Becker DJ, Dosch HM. Abnormal T-cell reactivities in childhood inflammatory demyelinating disease and type 1 diabetes. Ann Neurol. 2008;63:98–111. 10.1002/ANA.21244. 17932975 10.1002/ana.21244

[CR5] Bechtold S, Blaschek A, Raile K, Dost A, Freiberg C, Askenas M, Fröhlich-Reiterer E, Molz E, Holl RW. Higher relative risk for multiple sclerosis in a pediatric and adolescent diabetic population: analysis from DPV database. Diabetes Care. 2014;37:96–101. 10.2337/dc13-1414. 23990514 10.2337/dc13-1414

[CR6] Bending D, Zaccone P, Cooke A. Inflammation and type one diabetes. Int Immunol. 2012;24:339–46. 10.1093/intimm/dxs049. 22447815 10.1093/intimm/dxs049

[CR7] Botbol Y, Guerrero-Ros I, Macian F. Key roles of autophagy in regulating T-cell function. Eur J Immunol. 2016;46:1326–34. 10.1002/eji.201545955. 27151577 10.1002/eji.201545955 PMC5227655

[CR8] Brynedal B, Khademi M, Wallström E, Hillert J, Olsson T, Duvefelt K. Gene expression profiling in multiple sclerosis: a disease of the central nervous system, but with relapses triggered in the periphery? Neurobiol Dis. 2010;37:613–21. 10.1016/j.nbd.2009.11.014. 19944761 10.1016/j.nbd.2009.11.014

[CR9] Canet F, Díaz-Pozo P, Luna-Marco C, Fernandez-Reyes M, Vezza T, Marti M, Salazar JD, Roldan I, Morillas C, Rovira-Llopis S, Rocha M, Víctor VM. Mitochondrial redox impairment and enhanced autophagy in peripheral blood mononuclear cells from type 1 diabetic patients. Redox Biol. 2022;58:102551. 10.1016/j.redox.2022.102551. 36455476 10.1016/j.redox.2022.102551 PMC9713367

[CR10] Care D, Suppl SS. 2. Classification and diagnosis of diabetes: standards of medical care in diabetes-2021. Diabetes Care. 2021;44:S15–33. 10.2337/dc21-S002. 33298413 10.2337/dc21-S002

[CR11] Catarino D, Silva D, Guiomar J, Ribeiro C, Ruas L, Cardoso L, Paiva I. Non-immune-mediated versus immune-mediated type 1 diabetes: diagnosis and long-term differences—retrospective analysis. Diabetol Metab Syndr. 2020;12:4–9. 10.1186/s13098-020-00563-x. 32647539 10.1186/s13098-020-00563-x PMC7336466

[CR12] Chatterjee T, Pattanayak R, Ukil A, Chowdhury S, Bhattacharyya M. Autophagy protects peripheral blood mononuclear cells against inflammation, oxidative and nitrosative stress in diabetic dyslipidemia. Free Radic Biol Med. 2019;143:309–23. 10.1016/j.freeradbiomed.2019.07.034. 31369843 10.1016/j.freeradbiomed.2019.07.034

[CR13] Chou IC, Wang CH, Lin WD, Tsai FJ, Lin CC, Kao CH. Risk of epilepsy in type 1 diabetes mellitus: a population-based cohort study. Diabetologia. 2016;59:1196–203. 10.1007/s00125-016-3929-0. 27030312 10.1007/s00125-016-3929-0

[CR14] Cojocaru M, Cojocaru IM, Silosi I. Multiple autoimmune syndrome. Maedica. 2010;5:132–4. 21977137 PMC3150011

[CR15] Cotsapas C, Mitrovic M, Hafler D. Multiple sclerosis. Handb Clin Neurol. 2018;148:723–30. 10.1016/B978-0-444-64076-5.00046-6. 29478610 10.1016/B978-0-444-64076-5.00046-6

[CR16] Cox J, Mann M. MaxQuant enables high peptide identification rates, individualized p.p.b.-range mass accuracies and proteome-wide protein quantification. Nat Biotechnol. 2008;26:1367–72. 10.1038/nbt.1511. 19029910 10.1038/nbt.1511

[CR17] De Bondt M, Hellings N, Opdenakker G, Struyf S. Neutrophils: underestimated players in the pathogenesis of multiple sclerosis (ms). Int J Mol Sci. 2020;21:1–25. 10.3390/ijms21124558. 10.3390/ijms21124558 PMC7349048 32604901

[CR18] Deegan S, Saveljeva S, Gorman AM, Samali A. Stress-induced self-cannibalism: on the regulation of autophagy by endoplasmic reticulum stress. Cell Mol Life Sci. 2013;70:2425–41. 10.1007/s00018-012-1173-4. 23052213 10.1007/s00018-012-1173-4 PMC11113399

[CR19] Del Boccio P, Rossi C, di Ioia M, Cicalini I, Sacchetta P, Pieragostino D. Integration of metabolomics and proteomics in multiple sclerosis: from biomarkers discovery to personalized medicine. Proteomics—Clin Appl. 2016;10:470–84. 10.1002/prca.201500083. 27061322 10.1002/prca.201500083

[CR20] DiMeglio LA, Evans-Molina C, Oram RA. Type 1 diabetes. Lancet. 2018;391:2449–62. 10.1016/S0140-6736(18)31320-5. 29916386 10.1016/S0140-6736(18)31320-5 PMC6661119

[CR21] Ding X, Fang C, Li X, Cao YJ, Zhang QL, Huang Y, Pan J, Zhang X. Type 1 diabetes-associated cognitive impairment and diabetic peripheral neuropathy in Chinese adults: Results from a prospective cross-sectional study. BMC Endocr Disord. 2019;19:1–6. 10.1186/s12902-019-0359-2. 30917808 10.1186/s12902-019-0359-2 PMC6437981

[CR22] Endo Y, Kanno T, Nakajima T. Fatty acid metabolism in T-cell function and differentiation. Int Immunol. 2022;34:579–87. 10.1093/intimm/dxac025. 35700102 10.1093/intimm/dxac025

[CR23] Fidalgo M, Faria R, Carvalho C, Carvalheiras G, Mendonça D, Farinha F, da Silva BM, Vasconcelos C. Multiple autoimmune syndrome: clinical, immunological and genotypic characterization. Eur J Intern Med. 2023;116:119–30. 10.1016/J.EJIM.2023.06.020. 37385917 10.1016/j.ejim.2023.06.020

[CR24] Forga L, Bolado F, Goñi MJ, Tamayo I, Ibáñez B, Prieto C. Low serum levels of prealbumin, retinol binding protein, and retinol are frequent in adult type 1 diabetic patients. J Diabetes Res. 2016;2016:1–6. 10.1155/2016/2532108. 10.1155/2016/2532108 PMC5153501 28018921

[CR25] Galiero R, Caturano A, Vetrano E, Beccia D, Brin C, Alfano M, Di SJ, Epifani R, Piacevole A, Tagliaferri G, Rocco M, Iadicicco I, Docimo G, Rinaldi L, Sardu C, Salvatore T, Marfella R, Sasso FC. Peripheral neuropathy in diabetes mellitus : pathogenetic mechanisms and diagnostic options. Int J Mol Sci. 2023. 10.3390/ijms24043554. 36834971 10.3390/ijms24043554 PMC9967934

[CR26] Gonzalo H, Brieva L, Tatzber F, Jové M, Cacabelos D, Cassanyé A, Lanau-Angulo L, Boada J, Serrano JCE, González C, Hernández L, Peralta S, Pamplona R, Portero-Otin M. Lipidome analysis in multiple sclerosis reveals protein lipoxidative damage as a potential pathogenic mechanism. J Neurochem. 2012;123:622–34. 10.1111/j.1471-4159.2012.07934.x. 22924648 10.1111/j.1471-4159.2012.07934.x

[CR27] Gregory GA, Robinson TIG, Linklater SE, Wang F, Colagiuri S, de Beaufort C, Donaghue KC, Magliano DJ, Maniam J, Orchard TJ, Rai P, Ogle GD. Global incidence, prevalence, and mortality of type 1 diabetes in 2021 with projection to 2040: a modelling study. Lancet Diabetes Endocrinol. 2022;10:741–60. 10.1016/S2213-8587(22)00218-2. 36113507 10.1016/S2213-8587(22)00218-2

[CR28] Hamasaki M, Noda T, Baba M, Ohsumi Y. Starvation triggers the delivery of the endoplasmic reticulum to the vacuole via autophagy in yeast. Traffic. 2005;6:56–65. 10.1111/j.1600-0854.2004.00245.x. 15569245 10.1111/j.1600-0854.2004.00245.x

[CR29] Handel AE, Handunnetthi L, Ebers GC, Ramagopalan SV. Type 1 diabetes mellitus and multiple sclerosis: common etiological features. Nat Rev Endocrinol. 2009;5:655–64. 10.1038/nrendo.2009.216. 19884899 10.1038/nrendo.2009.216

[CR30] Hu Q, Che G, Yang Y, Xie H, Tian J. Histone deacetylase 3 aggravates type 1 diabetes mellitus by inhibiting lymphocyte apoptosis through the microRNA-296–5p/Bcl-xl axis. Front Genet. 2020. 10.3389/fgene.2020.536854. 33240312 10.3389/fgene.2020.536854 PMC7667129

[CR31] Huang J, Xiao Y, Xu A, Zhou Z. Neutrophils in type 1 diabetes. J Diabetes Investig. 2016;7:652–63. 10.1111/jdi.12469. 27181374 10.1111/jdi.12469 PMC5009125

[CR32] Jamil M, Wang W, Xu M, Tu J. Exploring the roles of basal transcription factor 3 in eukaryotic growth and development. Biotechnol Genet Eng Rev. 2015;31:21–45. 10.1080/02648725.2015.1080064. 26428578 10.1080/02648725.2015.1080064

[CR33] Jin CY, Yu SW, Yin JT, Yuan XY, Wang XG. Corresponding risk factors between cognitive impairment and type 1 diabetes mellitus: a narrative review. Heliyon. 2022;8: e10073. 10.1016/j.heliyon.2022.e10073. 35991978 10.1016/j.heliyon.2022.e10073 PMC9389196

[CR34] Kallaur AP, Reiche EMV, Oliveira SR, Simão ANC, de Pereira WLCJ, Alfieri DF, Flauzino T, de Proença CM, Lozovoy MAB, Kaimen-Maciel DR, Maes M. Genetic, immune-inflammatory, and oxidative stress biomarkers as predictors for disability and disease progression in multiple sclerosis. Mol Neurobiol. 2017;54:31–44. 10.1007/s12035-015-9648-6. 26732588 10.1007/s12035-015-9648-6

[CR35] Larochelle C, Alvarez JI, Prat A. How do immune cells overcome the blood–brain barrier in multiple sclerosis? FEBS Lett. 2011;585:3770–80. 10.1016/j.febslet.2011.04.066. 21550344 10.1016/j.febslet.2011.04.066

[CR36] Lassmann H. Multiple sclerosis pathology. Cold Spring Harb Perspect Med. 2018;8:1–16. 10.1101/cshperspect.a028936. 10.1101/cshperspect.a028936 PMC5830904 29358320

[CR37] Lehmensiek V, Süssmuth SD, Tauscher G, Brettschneider J, Felk S, Gillardon F, Tumani H. Cerebrospinal fluid proteome profile in multiple sclerosis. Mult Scler. 2007;13:840–9. 10.1177/1352458507076406. 17881397 10.1177/1352458507076406

[CR38] Lemus HN, Warrington AE, Rodriguez M. Multiple sclerosis: mechanisms of disease and strategies for myelin and axonal repair. Neurol Clin. 2018;36:1–11. 10.1016/j.ncl.2017.08.002. 29157392 10.1016/j.ncl.2017.08.002 PMC7125639

[CR39] Maahs DM, West NA, Lawrence JM, Mayer-Davis EJ. Epidemiology of type 1 diabetes. Endocrinol Metab Clin North Am. 2010;39:481–97. 10.1016/j.ecl.2010.05.011. 20723815 10.1016/j.ecl.2010.05.011 PMC2925303

[CR40] Malinowska M, Tokarz-Deptula B, Deptula W. Butyrophilins: an important new element of resistance. Cent Eur J Immunol. 2017;42:399–403. 10.5114/ceji.2017.72806. 29472819 10.5114/ceji.2017.72806 PMC5820976

[CR41] Mandel I, Paperna T, Miller A. Aberrant expression of the apoptosis-related proteins BAK and MCL1 in T cells in multiple sclerosis. J Neuroimmunol. 2012;244:51–6. 10.1016/j.jneuroim.2011.12.026. 22257632 10.1016/j.jneuroim.2011.12.026

[CR42] Marasco MR, Linnemann AK. β-cell autophagy in diabetes pathogenesis. Endocrinology. 2018;159:2127–41. 10.1210/en.2017-03273. 29617763 10.1210/en.2017-03273 PMC5913620

[CR43] Marrosu MG, Cocco E, Lai M, Spinicci G, Pischedda MP, Contu P. Patients with multiple sclerosis and risk of type 1 diabetes mellitus in Sardinia, Italy: a cohort study. Lancet. 2002;359:1461–5. 10.1016/S0140-6736(02)08431-3. 11988243 10.1016/S0140-6736(02)08431-3

[CR44] Masson GR. Towards a model of GCN2 activation. Biochem Soc Trans. 2019;47:1481–8. 10.1042/BST20190331. 31647517 10.1042/BST20190331 PMC6824675

[CR45] Melendez-Ramirez LY, Richards RJ, Cefalu WT. Complications of type 1 diabetes. Endocrinol Metab Clin North Am. 2010;39:625–40. 10.1016/j.ecl.2010.05.009. 20723824 10.1016/j.ecl.2010.05.009

[CR46] Misrielal C, Mauthe M, Reggiori F, Eggen BJL. Autophagy in multiple sclerosis: two sides of the same coin. Front Cell Neurosci. 2020. 10.3389/fncel.2020.603710. 33328897 10.3389/fncel.2020.603710 PMC7714924

[CR47] Mungan S, Guzel I, Demirdogen BC. Association between expanded disability status scale score and dietary antioxidant capacity in patients with multiple sclerosis. Braz J Med Biol Res = Rev Bras Pesqui Medicas e Biol. 2023;3:5. 10.1590/1414-431X2023E12776. 10.1590/1414-431X2023e12776 PMC10496758 37703109

[CR48] Muthulingam JA, Brock C, Hansen TM, Drewes AM, Brock B, Frøkjær JB. Disrupted white matter integrity in the brain of type 1 diabetes is associated with peripheral neuropathy and abnormal brain metabolites. J Diabetes Complications. 2022;36:108267. 10.1016/j.jdiacomp.2022.108267. 35905510 10.1016/j.jdiacomp.2022.108267

[CR49] Obeagu EI, Obeagu GU. Type 1 diabetes mellitus: roles of neutrophils in the pathogenesis. Medicine. 2023;102: e36245. 10.1097/MD.0000000000036245. 38115297 10.1097/MD.0000000000036245 PMC10727583

[CR50] Oreja-Guevara C, Martínez-Yélamos S, Eichau S, Llaneza MÁ, Martín-Martínez J, Peña-Martínez J, Meca-Lallana V, Alonso-Torres AM, Moral-Torres E, Río J, Calles C, Ares-Luque A, Ramió-Torrentà L, Marzo-Sola ME, Prieto JM, Martínez-Ginés ML, Arroyo R, Otano-Martínez MÁ, Brieva-Ruiz L, Gómez-Gutiérrez M, Rodríguez-Antigüedad A, Galán Sánchez-Seco V, Costa-Frossard L, Hernández-Pérez MÁ, Landete-Pascual L, González-Platas M, Meca-Lallana JE. Beyond lines of treatment: embracing early high-efficacy disease-modifying treatments for multiple sclerosis management. Ther Adv Neurol Disord. 2024;17:17562864241284372. 10.1177/17562864241284372. 39483817 10.1177/17562864241284372 PMC11526321

[CR51] Pagnin M, Dekiwadia C, Petratos S, Richardson SJ. Enhanced re-myelination in transthyretin null mice following cuprizone mediated demyelination. Neurosci Lett. 2022. 10.1016/J.NEULET.2021.136287. 34634393 10.1016/j.neulet.2021.136287

[CR52] Perais J, Agarwal R, Evans JR, Loveman E, Colquitt JL, Owens D, Hogg RE, Lawrenson JG, Takwoingi Y, Lois N. Prognostic factors for the development and progression of proliferative diabetic retinopathy in people with diabetic retinopathy. Cochrane Database Syst Rev. 2023. 10.1002/14651858.CD013775.pub2. 36815723 10.1002/14651858.CD013775.pub2 PMC9943918

[CR53] Pöll G, Griesenbeck J, Tschochner H, Milkereit P. Impact of the yeast S0/uS2-cluster ribosomal protein rpS21/eS21 on rRNA folding and the architecture of small ribosomal subunit precursors. PLoS ONE. 2023;18:1–21. 10.1371/journal.pone.0283698. 10.1371/journal.pone.0283698 PMC10062582 36996028

[CR54] Polman CH, Reingold SC, Banwell B, Clanet M, Cohen JA, Filippi M, Fujihara K, Havrdova E, Hutchinson M, Kappos L, Lublin FD, Montalban X, O’Connor P, Sandberg-Wollheim M, Thompson AJ, Waubant E, Weinshenker B, Wolinsky JS. Diagnostic criteria for multiple sclerosis: 2010 revisions to the McDonald criteria. Ann Neurol. 2011;69:292–302. 10.1002/ana.22366. 21387374 10.1002/ana.22366 PMC3084507

[CR55] Pompura SL, Hafler DA, Dominguez-Villar M. Fatty acid metabolism and T cells in multiple sclerosis. Front Immunol. 2022;13:1–15. 10.3389/fimmu.2022.869197. 10.3389/fimmu.2022.869197 PMC9116144 35603182

[CR56] Pozzilli V, Grasso EA, Tomassini V. Similarities and differences between multiple sclerosis and type 1 diabetes. Diabetes Metab Res Rev. 2022;38:1–5. 10.1002/dmrr.3505. 10.1002/dmrr.3505 PMC9285024 34651395

[CR57] Rispoli MG, Valentinuzzi S, De Luca G, Del Boccio P, Federici L, Di Ioia M, Digiovanni A, Grasso EA, Pozzilli V, Villani A, Chiarelli AM, Onofrj M, Wise RG, Pieragostino D, Tomassini V. Contribution of metabolomics to multiple sclerosis diagnosis, prognosis and treatment. Int J Mol Sci. 2021. 10.3390/ijms222011112. 34681773 10.3390/ijms222011112 PMC8541167

[CR58] Ruiz F, Vigne S, Pot C. Resolution of inflammation during multiple sclerosis. Semin Immunopathol. 2019;41:711–26. 10.1007/s00281-019-00765-0. 31732775 10.1007/s00281-019-00765-0 PMC6881249

[CR59] Safari-Alighiarloo N, Taghizadeh M, Tabatabaei SM, Shahsavari S, Namaki S, Khodakarim S, Rezaei-Tavirani M. Identification of new key genes for type 1 diabetes through construction and analysis of protein–protein interaction networks based on blood and pancreatic islet transcriptomes. J Diabetes. 2017;9:764–77. 10.1111/1753-0407.12483. 27625010 10.1111/1753-0407.12483

[CR60] Singh R, Cuervo AM. Autophagy in the cellular energetic balance. Cell Metab. 2011;13:495–504. 10.1016/j.cmet.2011.04.004. 21531332 10.1016/j.cmet.2011.04.004 PMC3099265

[CR61] Sun W, Shi Y, Yang J, Song X, Zhang Y, Zhang W, Zhou X. Transthyretin and retinol-binding protein as discriminators of diabetic retinopathy in type 1 diabetes mellitus. Int Ophthalmol. 2022;42:1041–9. 10.1007/s10792-021-02088-2. 34718910 10.1007/s10792-021-02088-2

[CR62] Toprak H, Yetis H, Alkan A, Filiz M, Kurtcan S, Aralasmak A, Saksu M, Cesur Y. Relationships of DTI findings with neurocognitive dysfunction in children with type 1 diabetes mellitus. Br J Radiol. 2016;89:11–5. 10.1259/bjr.20150680. 10.1259/bjr.20150680 PMC4986488 26728951

[CR63] Tyanova S, Temu T, Sinitcyn P, Carlson A, Hein MY, Geiger T, Mann M, Cox J. The perseus computational platform for comprehensive analysis of (prote)omics data. Nat Methods. 2016;13:731–40. 10.1038/NMETH.3901. 27348712 10.1038/nmeth.3901

[CR64] Tynyakov-Samra E, Auriel E, Levy-Amir Y, Karni A. Reduced ErbB4 expression in immune cells of patients with relapsing remitting multiple sclerosis. Mult Scler Int. 2011;2011:1–7. 10.1155/2011/561262. 10.1155/2011/561262 PMC3197252 22096639

[CR65] Ueda M. Transthyretin: its function and amyloid formation. Neurochem Int. 2022;155:105313. 10.1016/j.neuint.2022.105313. 35218869 10.1016/j.neuint.2022.105313

[CR66] Van Belle TL, Coppieters KT, Von Herrath MG. Type 1 diabetes: etiology, immunology, and therapeutic strategies. Physiol Rev. 2011;91:79–118. 10.1152/physrev.00003.2010. 21248163 10.1152/physrev.00003.2010

[CR67] van den Berg R, Hoogenraad CC, Hintzen RQ. Axonal transport deficits in multiple sclerosis: spiraling into the abyss. Acta Neuropathol. 2017;134:1–14. 10.1007/s00401-017-1697-7. 28315956 10.1007/s00401-017-1697-7 PMC5486629

[CR68] van Megen KM, Spindler MP, Keij FM, Bosch I, Sprangers F, van Royen-Kerkhof A, Nikolic T, Roep BO. Relapsing/remitting type 1 diabetes. Diabetologia. 2017;60:2252–5. 10.1007/s00125-017-4403-3. 28835984 10.1007/s00125-017-4403-3 PMC6448902

[CR69] Villoria-González A, Zierfuss B, Parzer P, Heuböck E, Zujovic V, Waidhofer-Söllner P, Ponleitner M, Rommer P, Göpfert J, Forss-Petter S, Berger J, Weinhofer I. Efficacy of HDAC inhibitors in driving peroxisomal β-oxidation and immune responses in human macrophages: implications for neuroinflammatory disorders. Biomolecules. 2023. 10.3390/biom13121696. 38136568 10.3390/biom13121696 PMC10741867

[CR70] von Herrath M, Sanda S, Herold K. Type 1 diabetes as a relapsing–remitting disease? Nat Rev Immunol. 2007;7:988–94. 10.1038/nri2192. 17982429 10.1038/nri2192

[CR71] Walton C, King R, Rechtman L, Kaye W, Leray E, Marrie RA, Robertson N, La Rocca N, Uitdehaag B, van der Mei I, Wallin M, Helme A, Angood Napier C, Rijke N, Baneke P. Rising prevalence of multiple sclerosis worldwide: Insights from the Atlas of MS, third edition. Mult Scler J. 2020;26:1816–21. 10.1177/1352458520970841. 10.1177/1352458520970841 PMC7720355 33174475

[CR72] Wang T, Wang ZY, Zeng LY, Gao YZ, Yan YX, Zhang Q. Down-regulation of ribosomal protein RPS21 inhibits invasive behavior of osteosarcoma cells through the inactivation of MAPK pathway. Cancer Manag Res. 2020;12:4949–55. 10.2147/CMAR.S246928. 32612383 10.2147/CMAR.S246928 PMC7323807

[CR73] Winer S, Astsaturov I, Cheung RK, Gunaratnam L, Kubiak V, Cortez MA, Moscarello M, O’Connor PW, McKerlie C, Becker DJ, Dosch H-M. Type I diabetes and multiple sclerosis patients target islet plus central nervous system autoantigens; nonimmunized nonobese diabetic mice can develop autoimmune encephalitis. J Immunol. 2001;166:2831–41. 10.4049/jimmunol.166.4.2831. 11160351 10.4049/jimmunol.166.4.2831

[CR74] Wiśniewski JR, Zougman A, Nagaraj N, Mann M. Universal sample preparation method for proteome analysis. Nat Methods. 2009;6:359–62. 10.1038/NMETH.1322. 19377485 10.1038/nmeth.1322

[CR75] Zhi W, Sharma A, Purohit S, Miller E, Bode B, Anderson SW, Reed JC, Steed RD, Steed L, Hopkins D, She JX. Discovery and validation of serum protein changes in type 1 diabetes patients using high throughput two dimensional liquid chromatography-mass spectrometry and immunoassays. Mol Cell Proteomics. 2011;10:1–10. 10.1074/mcp.M111.012203. 10.1074/mcp.M111.012203 PMC3226412 21900154

[CR76] Zoledziewska M, Costa G, Pitzalis M, Cocco E, Melis C, Moi L, Zavattari P, Murru R, Lampis R, Morelli L, Poddie F, Frongia P, Pusceddu P, Bajorek M, Marras A, Satta AM, Chessa A, Pugliatti M, Sotgiu S, Whalen MB, Rosati G, Cucca F, Marrosu MG. Variation within the CLEC16A gene shows consistent disease association with both multiple sclerosis and type 1 diabetes in Sardinia. Genes Immun. 2009;10:15–7. 10.1038/gene.2008.84. 18946483 10.1038/gene.2008.84

